# Antibiotics and Lipid-Modifying Agents: Potential Drug–Drug Interactions and Their Clinical Implications

**DOI:** 10.3390/pharmacy11040130

**Published:** 2023-08-19

**Authors:** Marios Spanakis, Danny Alon-Ellenbogen, Petros Ioannou, Nikolaos Spernovasilis

**Affiliations:** 1 Department Forensic Sciences and Toxicology, Faculty of Medicine, University of Crete, 71003 Heraklion, Greece; marspan@ics.forth.gr; 2 Computational Biomedicine Laboratory, Institute of Computer Science, Foundation for Research & Technology-Hellas (FORTH), 70013 Heraklion, Greece; 3 Department of Basic and Clinical Sciences, University of Nicosia Medical School, 2417 Nicosia, Cyprus; alon-ellenbogen.d@unic.ac.cy; 4 Department of Internal Medicine & Infectious Diseases, University Hospital of Heraklion, 71110 Heraklion, Greece; p.ioannou@uoc.gr; 5 Department of Infectious Diseases, German Oncology Center, 4108 Limassol, Cyprus

**Keywords:** antibiotics, lipid-modifying agents, drug–drug interactions, pharmaceutical care, antibiotic stewardship, good prescribing practices

## Abstract

Evidence-based prescribing requires taking into consideration the many aspects of optimal drug administration (e.g., dosage, comorbidities, co-administered drugs, etc.). A key issue is the administration of drugs for acute disorders that may potentially interfere with previously prescribed long-term medications. Initiating an antibiotic for an acute bacterial infection constitutes a common example. Hence, appropriate knowledge and awareness of the potential DDIs of antibiotics would lead to proper adjustments, thus preventing over- or under-treatment. For example, some statins, which are the most prescribed lipid-modifying agent (LMA), can lead to clinically important drug–drug interactions (DDIs) with the concurrent administration of antibiotics, e.g., macrolides. This review discusses the clinically significant DDIs of antibiotics associated with co-administrated lipid-lowering therapy and highlights common cases where regimen modifications may or may not be necessary.

## 1. Introduction

The rational use of medicines necessitates prescription according to the five ‘Rights’: the right medicine, the right patient, the right dose, the right duration, and at the right cost [1]. The prescribing of safe and effective medications is a multifactorial problem for physicians, especially in cases of patients with multimorbidity and chronic diseases that require adjunct therapies for acute conditions. Patients with multimorbidity are usually poly-medicated and are, thus, often at risk of polypharmacy (>5 simultaneously prescribed drugs) and potential drug–drug interactions (DDIs). This risk is increased in the face of acute conditions that require adjunct medications [2,3]. DDIs refer to the modulation of the pharmacological action and by extension to the potential alteration of clinical outcome from the co-administration of drugs that share common biological pathways. DDIs represent a major issue in optimal healthcare provision since the altered pharmacological profile of co-administered medications could raise the risk of adverse drug reactions (ADRs), treatment failure, and toxicity [4].

In the field of infectious diseases, a successful diagnosis of a pathogen is followed by proper treatment planning regarding the optimal therapy, which includes the choice of the most efficient antimicrobial agent at the correct dose and for the proper duration of administration [5]. Antimicrobial drugs are some of the most heavily prescribed medications in acute settings [6]. Antimicrobial agents, and especially antibiotics, display their pharmacological action by exclusively interfering in the cell cycle of bacteria, whereas they show minimum impact on human cells [7]. The discovery and use of antibiotics have certainly changed the way we deal with deadly infections caused by pathogens. On the other hand, the rising tide of bacterial resistance has led—albeit belatedly—the scientific community to take measures to better manage antibiotic use. Thus, in previous years, the gradual development of antibiotic stewardship programs and adherence to evidence-based practices and regulatory guidelines in prescribing led to better management or even a slight drop in antibiotic prescriptions in some countries in the Western world [8,9,10,11].

An additional issue in evidence-based clinical prescription practices is antibiotics being co-administered with other medications. Generally, most antibiotics have specific adverse events and side effects, while they also display few pharmacodynamic synergies. However, some groups of antibiotics have a greater interaction potential and increase the risk of ADRs of co-administered medications. Considering also that they are prescribed regularly, it is very important to understand and predict cases of potentially clinically significant DDIs between antibiotics and other co-administered drugs, especially in patient populations suffering from chronic diseases and under polypharmacy conditions. One such category is patients with hyperlipidemia who are receiving lipid-lowering therapy to control their cholesterol levels. A very frequent clinical concern regarding antibiotics involves the identification of cases where the co-administration with lipid-modifying agents (LMAs) is contraindicated or, more specifically, when LMAs should be withheld when treatment with antimicrobial agents is being initiated. The origin of this perception can be attributed to precautions that should be taken for statins which are usually the drug of choice regarding LMAs and they are often involved in DDIs leading to adverse drug events (ADEs) and side effects related to their use (i.e., rhabdomyolysis) [12].

The purpose of this review is to present the literature data available through MEDLINE as well as regulatory reports and information included within Summaries of Product Characteristics (SmPC) regarding the potential DDIs of antibiotics with LMAs and to discuss their clinical significance. To the best of our knowledge, except for statins, there is no summarized information available through the literature for other classes of LMAs as to their potential DDIs with antibiotics as well as the clinical significance. This review will focus on antibiotics for systematic use according to the anatomic therapeutic index (ATC-J01) and their potential DDIs with statins as well as with other LMAs (ATC-C10) and will not expand in detail on other antimicrobials such as antimycotics (ATC-J02), antimycobacterials (ATC-J03), and antivirals (ATC-J04).

## 2. Literature Research

We conducted a comprehensive literature review utilizing MEDLINE, regulatory reports, and SmPCs. The search was performed using a combination of Medical Subject Headings (MeSH) terms and keywords such as HMG-CoA reductase inhibitors (statins), simvastatin, lovastatin, atorvastatin, rosuvastatin, pravastatin, fluvastatin, pitavastatin, fibrates, bile acid sequestrants, ezetimibe, probucol, omega-3 fatty acids, new generation LMAs, mipomersen, bempedoic acid, proprotein convertase 9 inhibitors, antibiotics, erythromycin, clarithromycin, azithromycin, macrolides, fluoroquinolones, ciprofloxacin, pharmacokinetic interactions, pharmacodynamic interactions, adverse drug reactions, cytochrome P450, p-glycoprotein, and organic anion transporting polypeptides. The selection criteria included relevant English language, in vitro data, in vivo and clinical studies involving healthy volunteers, case reports, and population studies. By adopting this thorough approach, this review aimed to gather and synthesize relevant information on the topic of interest.

## 3. Pharmacological Mechanisms of Drug Interactions

**Figure 1:**
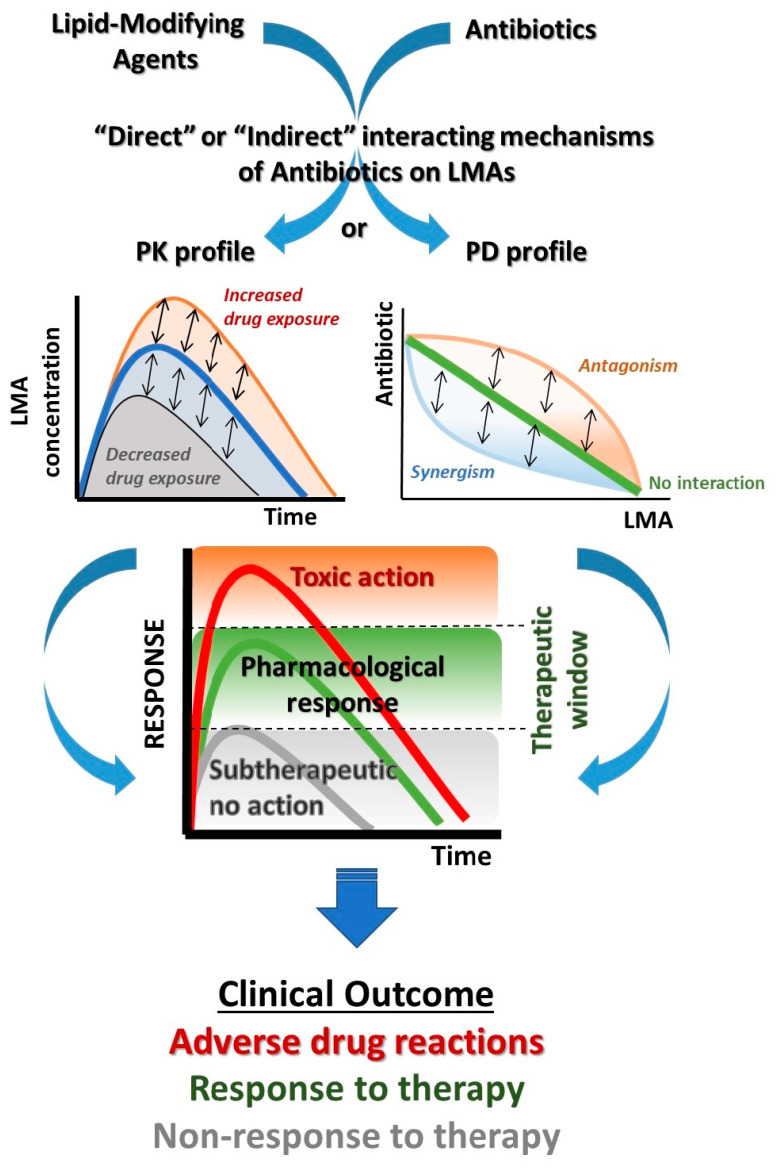
Potential drug interaction mechanisms between lipid-modifying agents and antibiotics and the impact on the pharmacological and clinical outcome for LMAs.

A potential DDI is described as an alteration in the exposure and/or response to a drug (victim drug) that has arisen because of co-administration with another drug (perpetrator drug). DDIs are clinically significant if the change in exposure or response can refer to either triggering an ADR or modulating the therapeutic effect of the victim drug outside its therapeutic window, resulting either in toxicity or subtherapeutic action (Figure 1) [4,13]. The pharmacological mechanisms of DDIs are related to pharmacokinetic (PK-DDIs0) processes and parameters of absorption, distribution, metabolism, and elimination (ADME) or pharmacodynamic (PD-DDIs) modulation in the primary or secondary biological targets.

PD-DDIs occur when two drugs affect the same physiological or biochemical pathway and the effects of one drug are altered additively, synergistically, or antagonistically by the presence of another drug at the site of action or in secondary tissues [14,15]. This results in either the enhancement or reduction in drug effects and/or an increase in the effect on secondary targets enhancing side effects. PD-DDIs are generally more complex phenomena than PK-DDIs because biological systems are networks with high complexity, rich in redundancies and feedback loops, whereas PK processes are more defined and are more easily quantifiable in terms of drug levels and elimination rate [16].

PK-DDIs during absorption refer to cases in which co-administered perpetrator drug(s) modulate the biopharmaceutical properties of a victim drug, changing either its dissolution profile as it passes through the gastrointestinal (GI) tract or its active transmembrane transport, which is regulated by efflux and influx transporters such as P-glycoprotein (P-gp) and organic anion transporter polypeptides (OATP) [17]. PK-DDIs that involve drug distribution are related mostly to plasma proteins (i.e., albumin or alpha-1-acid-glycoprotein) and the tissue binding affinities of co-administered drugs. For example, a perpetrator drug can lead a victim drug to be displaced from albumin’s binding sites—due to higher affinity—which may lead to higher concentrations of the unbound victim drug in plasma (i.e., warfarin) [18]. Similarly, some drugs can alter the distribution of other drugs by dysregulating active or passive transport mechanisms for certain barriers such as the blood–brain barrier, which may allow victim drugs to enter the brain easier and thus alter the distribution in the CNS [19]. Furthermore, the potential PK-DDIs due to metabolic procedures are probably the most common mechanism of a DDI and a point of focus even during drug development processes. Drugs, like all xenobiotics, are metabolized in the liver and in some other tissues by specific enzymes during two phases (phase I & phase II). The hallmarks of these would be the enzymes of the cytochrome P450 (CYP) family. The most often contributing CYPs in phase I drug metabolism are CYP2D6, CYP2C9, CYP2C19, CYP2E1, CYP1A1&2, CYP3A5, and, notably, CYP3A4. In phase II (conjugation reactions), the most notable participating agents are UDP-glucuronosyltransferases, sulfotransferases, N-acetyltransferases, glutathione S-transferases, and methyltransferases [20,21]. Hence, when two or more drugs are metabolized by the same enzyme, they can compete for the same metabolic pathway, or if a perpetrator drug is an inhibitor or inducer of a CYP, it can change the concentration profile of the other substrate (victim drug) and potentially modulate its efficacy. Finally, DDIs at the elimination phase occur mainly when active transport systems are involved. In these cases, drugs or their metabolites are excreted in urine (or through enterohepatic circulation through the bile into the feces) by specific transporters, such as P-gp or OATP systems. Thus, the modulation of the transport rate by a perpetrator can change the elimination rate of a victim drug.

Potential DDIs, either PK- or PD-related, can also be categorized based on their clinical significance or the likelihood of ADRs or side effects and the severity of the interaction with co-administered medications. In the case of antibiotics and co-administered medications such as LMAs, there are two main pathways that an interaction mechanism can occur. The first is related with the direct impact on any main or secondary PK or PD pathways of the co-administered drug, and the second (indirect) is through the modulation of the gut microflora by antibiotic therapy that can impact the absorption processes of several medications that are administered per os [22,23,24]. The DDI’s outcome can vary depending on the extent of pharmacological modulation, potentially resulting in adverse drug events, unchanged pharmacological response, or subtherapeutic response. (Figure 1). Contemplating the accumulated evidence derived from experts’ opinions, in silico, in vitro and in vivo data, clinical studies, systematic reviews and meta-analyses, and reports from regulatory authorities that update the Summary of Product Characteristics, potential DDIs can be categorized considering clinical significance as “Serious-Use alternative” (SUA), “Use with caution-Monitor” (Monitor), and “Moderate-Minor” (MM) DDIs [4,25,26,27].

## 4. Antibiotic and LMA Prescription Trends in EU Countries

According to the annual epidemiological report of 2021 published by the European Center for Disease Prevention and Control, in community sectors of all EU countries, the population-weighted mean consumption of antibacterial agents for systemic use (tetracyclines, amphenicols, beta-lactams, sulfonamides, macrolides, aminoglycosides, and quinolones) was calculated to be at around 15 defined daily doses (DDD) per 1000 inhabitants per day (range 7.2–24.3), whereas for the hospital sector, it was 1.4 DDD per 1000 inhabitants per day (range: 0.7–2.2) [10]. Overall, considering that this report includes the COVID-19 era which raised concerns regarding a potential increase in antimicrobial prescriptions, the consumption of antibiotics seems not to have increased significantly during the pandemic and actually stabilized or even decreased in some countries, possibly due to antibiotic stewardship programs involving stricter policies of administration, among other reasons [28].

LMAs are widely recommended for the prevention of cardiovascular diseases (CVDs) due to atherosclerotic events. Based on a recent cross-sectional ecological study from 83 countries, the annual use of LMAs expressed as the compound annual growth rate (CAGR) increased at 4.13% from 2008 to 2018, reflecting mostly the increase in statin administration (CAGR 5.19%), ezetimibe, and PGSK9 inhibitors, whereas fibrate, niacin, omega-3 fatty acid, and bile acid sequestrant prescription rates declined [29]. The use of LMAs varies widely according to region and country. Generally, regions of the developed world (Europe, North America, and Oceania) showed higher levels of administration compared to other regions (i.e., Asia, Latin America, and Africa). The USA, Greece, and France had the highest consumption of LMAs measured in standard units per 1000 inhabitants. The results from recent epidemiological data suggest the prevalence of hypercholesterolemia in Greece to be over 50% in the adult population. Approximately 15% of these patients will be treated with LMAs, rising with age. In view of the type of LMAs, statins are the drug class of choice (>90% of monotherapy cases), followed by the addition of ezetimibe (9%) [30].

## 5. Antibiotic and LMA DDIs: Mechanisms and Clinical Significance

LMAs exert their pharmacological effects through various mechanisms, targeting key pathways involved in lipid metabolism and mainly on cholesterol synthesis, absorption, and metabolism [31]. Regarding their PK properties, they exhibit variable PK characteristics related to their chemical structure and physicochemical properties. In addition, their PK properties and by extension their pharmacological action are associated with specific transport and metabolic systems as well as genetic variations that are often observed for those pathways. For example, statins enter hepatocytes through active uptake transport from OATPs (i.e., OATP1B1 and OATP1B3) [32,33,34,35,36]. Table 1 summarizes the association of OATPs, P-gp, and CYP enzymes with several examples of drugs belonging to different LMA classes.

### 5.1. HMG CoA Reductase Inhibitors and Antibiotics

HMG-CoA reductase inhibitors are undoubtedly the main issue when it comes to co-administration with antibiotics. Statins are effective modulators of lipid metabolism due to the reversible and competitive inhibition of 3-hydroxy-3-methylglutaryl coenzyme A (HMG-CoA) reductase. The drop in LDL cholesterol levels associated with their use has led to a decrease in both cardiovascular morbidity and mortality [37]. Although they act on the same target, pharmacological differences between them should be taken into consideration when a statin is selected. As stated earlier, drug transporters, such as OATPs and P-gp, along with CYP-metabolizing enzymes, and their genetic variations significantly influence the pharmacokinetics of statins (Table 1). Especially for OATPs, their contribution to the statins’ pharmacological profile has been shown to be crucial, and pharmacogenomic data have been proposed to be important regarding the expected clinical outcome. For example, the single nucleotide polymorphism (c.521T > C, p.Val174Ala) in the SLCO1B1 gene reduces the transport capacity of OATP1B1, leading to elevated plasma levels of simvastatin acid and elevating the risk of simvastatin-induced myopathy [17,35]. Hence, the incorporation of pharmacogenomic testing for statin-related variants has the potential to enhance the safety and effectiveness of statin therapy (mostly for simvastatin) while mitigating ADR risks and aligning with evidence-based prescribing practices that are tailored to individual patients [33,36]. Most statins, except for pravastatin, rosuvastatin, and fluvastatin, are subject to first-pass metabolism by CYP3A4, and their bioavailability varies from ~5% for simvastatin to 60% for cerivastatin. The impact of the first-pass effect and low bioavailability is important since small changes can result in larger variations in drug exposure compared to drugs with medium or high bioavailability [38,39]. All statins have a protein binding ≥90% (except pravastatin which has ~50%) and are all affected by alterations in renal function (except for atorvastatin and fluvastatin). Moreover, their CYP metabolites (except for fluvastatin) contribute to the lipid-lowering effects [40]. Due to their utter prevalence as effective LMAs and their PK characteristics, statins are often involved in potential DDIs as victim drugs. Their side effects involve mostly muscles (e.g., myopathies, rhabdomyolysis) and the liver (e.g., elevated liver enzymes) [12,37,41]. Hence, it is often recommended to adjust the dose when a CYP3A4 inhibitor (or CYP2C9 for rosuvastatin) is about to be co-administered.

**Figure 2:**
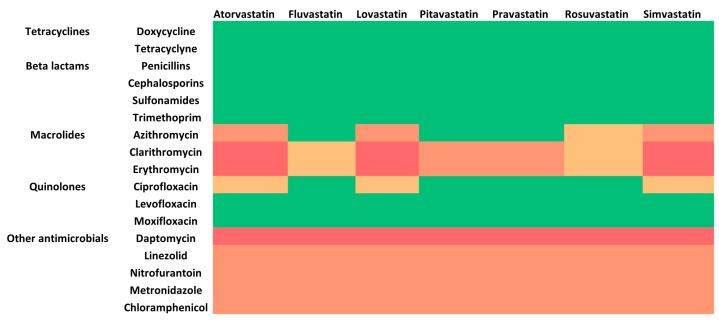
A heat-map infographic of potentially clinically significant DDIs between statins and antibiotics (red: avoid co-administration; orange: dose adjustment or monitor; yellow: moderate–minor; green: no DDIs).

Regarding antibiotics, there are some noteworthy potential DDIs that should be considered when the patient is under statin treatment (Figure 2) [42]. The issue is raised mostly for macrolides, which are known CYP3A4 inhibitors, and especially for erythromycin and clarithromycin, for which the co-administration should be avoided [43,44,45]. Similarly to macrolides, fluoroquinolones such as ciprofloxacin and levofloxacin can inhibit the CYP3A4 enzyme and P-gp, leading to increased bioavailability for some statins, such as atorvastatin, simvastatin, and lovastatin. This can increase the risk of statin-induced myopathy or rhabdomyolysis; thus, caution and monitoring are required [46]. Similarly to macrolides and fluoroquinolones, lefamulin, a new antibiotic for the treatment of community-acquired bacterial pneumonia, and streptogramins (i.e., quinupristin/dalfopristin) have shown inhibitory potential against CYP3A4, and, therefore, PK-DDIs with statin substrates of CYP3A4 can potentially occur [47,48]. For other statins, such as rosuvastatin and fluvastatin, the potential for DDIs through CYP inhibition is minimal—if not absent. Table 2 describes the dose adjustment recommendations for patients under statin therapy based on data available thus far [42]. Nevertheless, in the context of optimum pharmaceutical care practices, the patient should always be warned to report any symptoms of myopathy or other statin-related ADRs to their healthcare provider.

Except for the most common cases of potential PK-DDIs, PD-DDIs exist for statins and certain antibiotics. For example, there is a potential risk for myopathy in patients receiving treatment with a statin in combination with daptomycin, but the pharmacological mechanisms are not clarified with regards to whether it is the result of a DDI synergy or involves different pathways that lead to the same ADR. However, considering the known risks and unless there is an insistent need to continue therapy, statins are to be discontinued if daptomycin therapy is to be initiated [49,50]. Moreover, if linezolid, nitrofurantoin, metronidazole, or chloramphenicol are to be administered in a patient under statin treatment, caution is advised due to the shared side effect of peripheral neuropathy with symptoms such as burning, tingling, pain, or numbness in the hands and feet. The same precaution should be considered when isoniazid is about to be administered. These potential PD-DDIs can be a result of the impending synergistic effects of both drugs in their secondary targets [51,52,53]. Apart from the cases which were mentioned before and are well documented in the literature, the co-administration of statins with other antibiotic classes (tetracyclines, beta-lactams, sulfonamides, trimethoprim, etc.) does not generally require further adjustments (Figure 2).

### 5.2. Fibrates and Antibiotics

The pharmacological mechanism of fibrates, although not completely explained, is thought to involve the activation of peroxisome-proliferator-activated receptors (PPAR-alpha), leading to the regulation of gene expression and modulation of lipid metabolism and resulting in a reduction in triglyceride levels and improvement of lipid profiles in patients with dyslipidemia. They are usually prescribed as LMAs in atherogenic dyslipidemia characterized by high triglyceride and low HDL-C levels and, in this case, they son, are thought to be a good alternative to statins, especially when the dyslipidemia is associated with other metabolic syndromes (i.e., diabetes type II) [54]. Fibrates are generally well tolerated and, although they may increase creatinine and homocysteine levels, they are not associated with a risk for renal failure. Most fibrates (i.e., clofibrate, gemfibrozil, fenofibrate) are bound to albumin and metabolized by the hepatic CYPs, primarily by CYP2C19 and to a much lesser extent by CYP3A4, whereas gemfibrozil inhibits CYP2C9. All fibrates are primarily excreted via the kidneys and display some increase in plasma half-life in individuals with severe renal impairment [55].

As to the potential DDIs of fibrates, the evidence suggests that there are not any clinically significant DDIs, and no adjustments are needed in cases where an antibiotic therapy needs to be initiated. This applies for any antibiotic class. The only prescribing considerations should be focused on co-administration with highly protein-bound drugs, co-administration with gemfibrozil, which inhibits the metabolism of CYP2C9 substrates, and co-administration with statins, which raise the risk for muscle-related adverse events [56].

### 5.3. Bile Acid Sequestrants and Antibiotics

Bile acid sequestrants, including cholestyramine, colestipol, and colesevelam, exert their therapeutic effects through binding to bile acids in the GI tract and forming insoluble complexes that are not absorbed but lead to fecal elimination. This stimulates the liver to upregulate bile acid synthesis from cholesterol, resulting in a reduction in circulating cholesterol, specifically LDL cholesterol.

There are some concerns that bile acid sequestrants can interact with some antibiotics, such as tetracyclines and fluoroquinolones, in the GI tract and alter their absorption [57,58]. This potential PK-DDI has been demonstrated through some in vivo experiments in mice and can be of moderate–minor significance and easily resolved by creating a 4–6-h gap between the two administrations. Moreover, it has been proposed that co-administration may be beneficial in some cases to avoid the emergence of resistant bacteria colonizing the GI tract, for example, during daptomycin therapy [59].

### 5.4. Other LMAs and Antibiotics

The most often co-administered LMA with a statin is ezetimibe, and there are numerous medicinal products which contain ezetimibe combined with simvastatin or atorvastatin. Ezetimibe, which is also a standalone treatment approach for elevated cholesterol levels, is an inhibitor of intestinal cholesterol absorption by selectively blocking the NPC1L1 protein (Niemann-Pick C1-like 1 protein), which is located in the gut lumen, thus further contributing to the antilipidemic effect of statins [60]. Ezetimibe is poorly absorbed and is not metabolized by CYP450 but undergoes extensive glucuronidation metabolism in the intestine. Thus, due to its pharmacological properties, no DDIs have been described; hence, co-administration with antibiotics is not expected to produce any risk for ADRs [61]. The only concerns would be for medicinal products containing ezetimibe with simvastatin or atorvastatin, but these cases are related with potential DDIs with these specific statins and not ezetimibe.

As to other LMAs, probucol, which seems to act through the increased secretion of bile acids and blockade of LDL synthesis, received attention for its effectiveness for familial hypercholesterolemia. Its use has been limited due to its negative effect on HDL levels and the potential for causing arrhythmias. Hence, another consideration for probucol is that its co-administration with drugs that predispose to QT prolongation, such as macrolides and quinolones, should be avoided or occur with caution [62,63].

Omega-3 fatty acids, which are dispensed from pharmacies as fish oil supplements, are known for their potential health benefits for the prevention of atherosclerosis through the reduction in blood triglyceride levels and a positive impact on HDL [64]. Considering co-administration with antibiotics, there are no significant known DDIs (recorded or theoretically) between omega-3 fatty acids and antibiotics [65].

### 5.5. New Generation LMAs and Potential Interactions with Antibiotics

In the previous 10–20 years, there was substantial progress toward the discovery of novel lipid-lowering agents. The further understanding of biological processes that contribute to high cholesterol levels along with the promotion of novel biomedical molecules such as monoclonal antibodies, interfering RNA, peptide mimics, etc., have shown promising results as the next generation of medicinal molecules for the prevention and treatment of atherosclerosis and the related CVDs [66,67]. These novel biomedical products, among others, have the advantage of limited potential DDIs, including potential DDIs with antibiotics, at least till evidence from clinical trials and post-marketing studies suggests otherwise.

Mipomersen (Kynamro^®^) is an antisense oligonucleotide (ASO) inhibitor administered through subcutaneous injection to treat homozygous familial hypercholesterolemia, and it is approved in the US but not in the EU. Considering any clinically significant DDIs, caution or avoidance should be exercised when mipomersen is used with other medications known to have the potential for hepatotoxicity, such as tetracyclines (i.e., doxycycline, minocycline, tetracycline, etc.) or macrolides (i.e., clarithromycin or erythromycin) [68].

Bempedoic acid (Nexletol^®^) is an ATP citrate lyase inhibitor administered to patients with primary hypercholesterolemia and to patients with mixed dyslipidemia that are also on a low-fat diet and usually under statin co-administered therapy [66]. In vitro metabolic interaction studies suggest that bempedoic acid, as well as its active metabolite and glucuronide forms, are not metabolized by and do not inhibit or induce cytochrome P450 enzymes. They are not substrates for transporter proteins, although they both weakly inhibit OATP1B1 and OATP1B3, which may result in elevated concentrations of relative substrates (including some statins) [69]. Regarding antibiotics, there is a potentially clinically significant PD-DDI due to the potential synergy when combined with other drugs that increase the risk for tendinitis or tendon rupture, such as quinolone antibiotics (i.e., ciprofloxacin).

Proprotein convertase subtilisin/kexin type 9 (PCSK9) inhibitors alirocumab (Praluent^®^) and evolocumab (Repatha^®^) are a new class of LMA drugs for the treatment of familial hypercholesterolemia for patients who did not reach their target LDL on previous combination therapy of statin + ezetimibe and for those intolerant of statins [66]. They are monoclonal antibodies that act by blocking the activity of PCSK9, which plays a role in cholesterol homeostasis. The PCSK9 pathway has attracted a lot of attention in general, with several state-of-the-art approaches under development, including antisense oligonucleotide (siRNA), peptide mimics, and monoclonal antibodies. Inclisiran (Leqvio^®^) is a PCSK9-targeting short interfering RNA (siRNA) administered as a subcutaneous injection typically every 3 months and often combined with other LMAs (e.g., statins + ezetimibe) when LDL levels are not adequately controlled. No clinically significant DDIs should be expected when antibiotic therapy is about to be initiated [67].

## 6. Discussion

The rational use of medicines requires physicians to be aware and vigilant regarding the proper medication regimen and make necessary adjustments [70]. Reliable evaluation methods are essential for evidence-based clinical decision support in DDIs that ensures valid assessment and helps healthcare professionals make informed decisions, optimizing medication therapy and enhancing patient safety [4,71,72]. A further understanding of the implicated PK or PD mechanisms that can potentially lead to DDIs and ADRs has alerted physicians to improve the way they prescribe certain medications, especially for chronic diseases. A representative example is the prescription of statins, where accumulating evidence in recent years has facilitated the characterization of ADRs resulting from DDIs, such as skeletal muscle toxicity. This knowledge has enabled the development of specific approaches to minimize ADRs without compromising therapeutic benefits [73,74].

A critical issue from the point of best prescription practices is that of acute conditions, such as in the cases of acute infections and the need for antimicrobial therapy in patients with chronic diseases for which multiple medicines may be administered [75]. The scientific community and the regulatory committees have very quickly and pointily provided certain guidance for specific antimicrobials that are known to interact with various medications, including statins [42,76]. For example, antifungal agents (ketoconazole, fluconazole, itraconazole) are known inhibitors of CYP3A4; thus, per os co-administration with drugs that are substrates of CYP3A4 (i.e., simvastatin, lovastatin, atorvastatin) may lead to clinically significant DDIs and ADRs due to increased drug exposure that requires either treatment cessation or dose adjustment [42,77,78]. Another antimicrobial with a well-documented mechanism is rifampin, an antituberculosis agent. Rifampin causes the induction of CYP3A4 and an increase in P-gp levels, accelerating the metabolism of all drugs that are substrates of these two biochemical pathways, including certain statins [79,80]. Rifampin is usually administered for periods of months; thus, specific instructions exist when rifampicin therapy is to be initiated, including dose adjustment for certain patients [81]. It is possible that these cases of potentially clinically significant DDIs and the relative guideline gave rise to an often unreasonable generalization and the cultivation of a tendency to withhold the administration of any antilipidemic agent when an antimicrobial drug is administered regardless of which class it is, antibiotic, antimicrobial, or antifungal.

**Figure 3:**
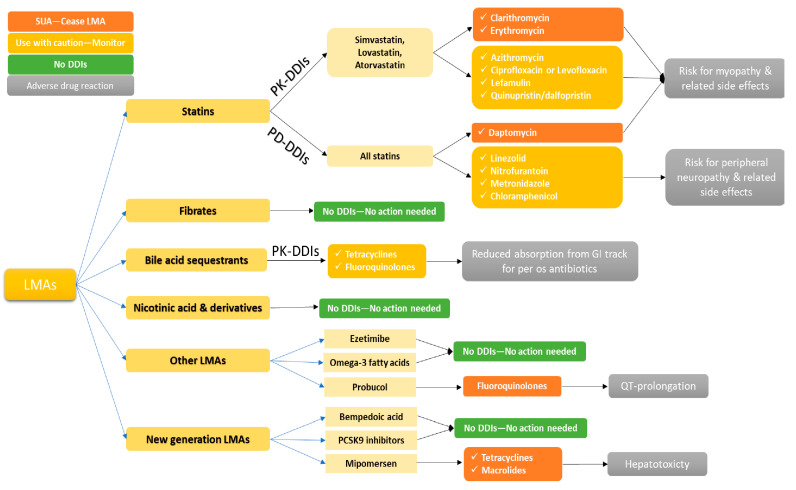
Examples of pharmacokinetic (PK) or pharmacodynamic (PD) drug–drug interactions (DDIs) of lipid-modifying agents (LMAs) and antibiotics along with potential clinical outcome (SUA: serious—use alternative).

**Figure 4:**
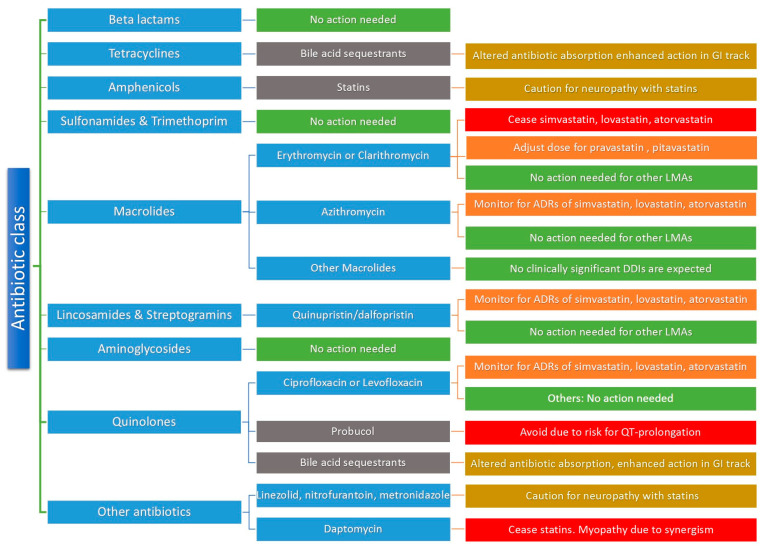
Clinical considerations towards optimum prescribing practices when an antibiotic therapy is about to be initiated in a patient under treatment with LMAs.

The co-administration of commonly prescribed antibiotics is generally safe in terms of potential clinically significant DDIs with LMAs. Figure 3 and Figure 4 provide a general approach for clinical considerations for the most common antibiotic classes. Figure 3 illustrates potential DDIs for each LMA category, the pharmacological mechanism involved, and the possible ADRs that may be observed clinically. Figure 4 describes clinical considerations when an antibiotic therapy is about to be initiated in a patient under treatment with LMAs. In general, beta-lactams do not require any treatment modifications for any LMA. The same can be said for sulfonamides, trimethoprim, and aminoglycosides, as currently available evidence suggests that any potential mechanisms cannot lead to clinically significant DDIs with LMAs [82,83,84]. Regarding tetracyclines, no specific precautions are needed except in cases where bile acid sequestrants are used, which can impact tetracyclines’ absorption or lead to enhanced activity in the gastrointestinal tract, which may impact gut microflora [57]. If macrolides are prescribed, lipid-lowering therapy should be carefully examined. Co-administration with statins should be examined by first establishing which statin is prescribed. If the patient is under treatment with lovastatin, atorvastatin, or simvastatin, co-administration should be avoided, or statin therapy should be discontinued for a short period. If pitavastatin or pravastatin is administered, a dose adjustment can be considered, whereas fluvastatin or rosuvastatin can be continued [44]. Azithromycin is a less potent CYP3A4 inhibitor than other macrolides, and therefore a statin dose adjustment may be required only if the patient is prescribed lovastatin, atorvastatin, or simvastatin, while no action is needed for other statins [85]. The available evidence suggests that the rest of the macrolides pose a minimum risk for DDIs, and no adjustments are needed. Similar to macrolides, lincosamides and streptogramins (e.g., clindamycin and quinupristin/dalfopristin, respectively) can lead to DDIs due to moderate inhibition of CYP3A4; thus, caution is needed with statin co-administration. Generally, for these groups of antibiotics, and mostly for these specific ones, most PK-DDIs are related to simvastatin, lovastatin, and atorvastatin (Figure 2). These cases may require monitoring and dose adjustments or, in extreme cases, discontinuing LMA administration for the active treatment period with the antibiotic therapy. In this respect, another important antibiotic class that should be examined for co-administration with LMAs is quinolones. The available evidence suggests that the co-administration has no clinical significance, except in cases where ciprofloxacin or levofloxacin are administered with simvastatin, lovastatin, or atorvastatin, which raise the risk for rhabdomyolysis [86,87].

As to other LMAs and clinically significant DDIs with quinolones, probucol should be avoided due to the risk of arrhythmias from QT-prolongation, whereas, similarly to tetracyclines, bile acid sequestrants may lead to reduced quinolone absorption and enhanced action in the GI tract, which may impact the gut microflora. For new generation LMAs, precaution is needed when mipomersen is used due to the increased risk for hepatotoxicty when administered with tetracyclines or macrolides. Finally, if antimicrobial treatment requires the administration of linezolid, nitrofurantoin, or metronidazole, caution is advised to avoid any potential synergy that may lead to peripheral neuropathy with statins, whereas the co-administration with daptomycin is suggested to be avoided due to an elevated risk of myopathy [50,51,52].

This work focuses mostly on cases in which special precautions should be considered within the context of evidence-based medicine to avoid any clinically significant DDIs between antibiotics and LMAs. It is noteworthy to point out that while the co-administration of antibiotics with statins raises concerns about potential DDIs, recent evidence suggests that certain statins may also possess antimicrobial properties. [88]. Statins’ antimicrobial action involves enhancing the host’s defense mechanisms through the stimulation of antimicrobial peptides and phagocytosis by immune cells while inhibiting pathological inflammation pathways and the production of essential components of bacterial cell membranes [89]. Further research is still underway to fully explore the potential antimicrobial and anti-inflammatory properties of statins and their pharmacodynamic potential [90]. Currently, simvastatin and atorvastatin are considered the most promising candidates for repurposing as novel adjuvant antimicrobials, particularly for addressing antimicrobial resistance in the future [91]. Hence, even in the context of DDIs and the current guidance for vigilance for simvastatin and atorvastatin, it may prove in the future that alternative combinations of repurposed statins with antibiotics may be helpful instead of unwanted, e.g., atorvastatin with metronidazole for anti-blastocystis therapy [90,92].

The above clinical considerations can provide valuable guidance for physicians when prescribing antibiotic therapies in patients under LMA treatment (Figure 4). However, it is important to note that other factors, such as dosage, comorbidities, and other co-administered medications, can also influence the risk of DDIs. For example, drug-induced kidney injury is a frequent adverse event that has been associated with antibiotics and may impact the PK profile of several drugs or their metabolites that are eliminated through the kidneys [93]. Therefore, prescribing physicians should carefully weigh the benefits and risks of using these medications concurrently, especially in patients with underlying medical conditions or those taking multiple medications. Patients with liver or kidney disease may be particularly vulnerable to DDIs and adverse effects. Moreover, physicians should be vigilant about potential DDIs with other groups of medicines, not just LMAs, when initiating antibiotic treatment for these patient cohorts. For example, macrolides’ and quinolones’ capability for QT-prolongation increases the risk of arrhythmias when they are co-administered with drugs other than LMAs that can promote the same side effect [94]. A personalized approach, based on careful evidence-based medicine, is necessary for each patient to optimize the outcomes and minimize the risk of DDIs. By taking these factors into consideration, physicians can improve prescription practices and ultimately enhance patient safety and therapy outcomes.

## 7. Conclusions

Towards optimum healthcare provision, DDIs are an important issue in clinical practice, especially when prescribing multiple medications to a patient with comorbidities. Considering antibiotics and LMAs, while there are a few possible clinically significant DDIs, most often prescribed cases of co-administrations are safe and do not require any adjustments or precautions other than those typically applied in terms of optimum pharmaceutical care. Overall, it is important for healthcare professionals to consider the individual patient’s medication regimen, medical history, and potential risk factors for adverse events when determining the appropriate treatment plan. Hence, a comprehensive and personalized approach is essential for successful healthcare provision in acute disorders, such as an infection, without disrupting the treatment of chronic conditions, such as cardiovascular diseases.

## Figures and Tables

**Table 1:** Transporters and metabolic enzymes that contribute to pharmacokinetics of LMAs.

LMA ^1^	OATP1B1 ^2^	OATP1B3 ^2^	P-gp ^3^	CYP3A4 ^4^	CYP2C9 ^4^
Atorvastatin	Substrate	Substrate	Substrate	Substrate	-
Fluvastatin	Substrate	Substrate	-	-	Substrate
Lovastatin	Substrate	Substrate	Substrate	Substrate	Substrate
Pitavastatin	Substrate	Substrate	Substrate	Substrate	-
Pravastatin	Substrate	Substrate	-	Substrate	Substrate
Rosuvastatin	Substrate	Substrate	Substrate	Substrate	Substrate
Fenofibrate	Inhibitor	-	Inhibitor	Substrate	Substrate
Gemfibrozil	Inhibitor	-	Inhibitor	Substrate	Inhibitor
Bezafibrate	Inhibitor	-	-	Substrate	Substrate
Ciprofibrate	-	-	-	Substrate	Substrate
Ezetimibe	Substrate	Substrate	Substrate	-	-

^1^ LMA: lipid-modifying agent, ^2^ OATP: organic anion transporter, ^3^ P-gp: P-glycoprotetin, ^4^ CYP: cytochrome P450).

**Table 2:** Dose adjustment recommendations for statins when antibiotic therapies are to be initiated.

	Atorvastatin (10–80 mg/day)	Fluvastatin (40–80 mg/day)	Lovastatin (20–80 mg/day)	Pitavastatin (1–4 mg/day)	Pravastatin (20–80 mg/day)	Rosuvastatin (5–40 mg/day)	Simvastatin (10–80 mg/day)
**Azithromycin**	Sustain and monitor	-	Sustain and monitor	-	-	-	Sustain and monitor
**Clarithromycin**	Cease	Sustain and monitor	Cease	Adjust to 1 mg/day	Limit to 40 mg/day	Sustain and monitor	Cease
**Erythromycin**							
**Ciprofloxacin**	Sustain and monitor	-	Sustain and monitor	-	-	-	Sustain and monitor
**Daptomycin**	Cease						
**Linezolid** **Nitrofurantoin Metronidazole Chloramphenicol**	Sustain and monitor						

## Data Availability

Not applicable.
